# Whole-genome sequencing and comparative genomic analysis of *Escherichia coli* O91 strains isolated from symptomatic and asymptomatic human carriers

**DOI:** 10.1186/s13099-016-0138-9

**Published:** 2016-11-11

**Authors:** Taesoo Kwon, Young-Seok Bak, Young-Hee Jung, Young-Bin Yu, Jong Tae Choi, Cheorl-Ho Kim, Jung-Beom Kim, Won Kim, Seung-Hak Cho

**Affiliations:** 1 Interdisciplinary Program in Bioinformatics, Seoul National University, 1 Gwanak-ro, Gwanak-gu, Seoul, 151-742 Republic of Korea; 2 Department of Emergency Medical Service, Sun Moon University, Asan-si, Chungcheongnam-do, 31460 Republic of Korea; 3 Division of Antimicrobial Resistance, Center for Infectious Diseases, Korea National Institute of Health, Cheongju, 363-951 Republic of Korea; 4 Department of Biomedical Laboratory Science, College of Medical Science, Konyang University, Daejeon, 302-832 Republic of Korea; 5 Department of Biomedical Laboratory Science, Kyungdong University, 815 Gyeonhwon-ro, Munmak-eup, Wonju-si, Gangwon-do 26495 Republic of Korea; 6 Glycobiology Unit, Department of Biological Science, Sungkyunkwan University and Samsung Advanced Institute for Health Sciences and Technology (SAIHST), 2066 Seobu-ro, Suwon, 16419 Republic of Korea; 7 Department of Food Science and Technology, Sunchon National University, Sunchon, Jeonnam 540-950 Republic of Korea; 8 Division of Enteric Diseases, Center for Infectious Diseases, Korea National Institute of Health, Cheongju, 363-951 Republic of Korea

**Keywords:** Shiga-like toxin-producing *Escherichia coli* O91, Draft genome, Type I fimbriae, *fimD*, Truncated protein

## Abstract

**Background:**

The Shiga toxin–producing *Escherichia coli* (STEC) O91:H21 strains NCCP15736 and NCCP15737 were isolated during a single outbreak in Korea, NCCP15736 from a symptomatic carrier and NCCP15737 from an asymptomatic carrier. To investigate genomic differences between the two strains, we performed whole-genome sequencing of both strains and conducted a comparative genomic analysis.

**Results:**

Using the Illumina HiSeq 2000 platform and Rapid Annotation using the Subsystem Technology (RAST) server, whole-genome sequences of NCCP15736 and NCCP15737 were obtained and annotated. Phylogenetic analysis of ten *E. coli* strains showed that NCCP15736 and NCCP15737 are evolutionarily close. The two strains were found to be most close to *E. coli* O91:NM str. 2009C-3745. The genomic comparison showed that the *fimD* gene of NCCP15737 is truncated and that the truncation could underlie the defects in infection and pathogenicity of NCCP15737. The two strains showed the same virulence factor profiles, and we identified 25 virulence factors from NCCP15736 and NCCP15737, respectively. We identified ten and nine phage-associated regions in the NCCP15736 and NCCP15737 genomes, respectively; the two strains share five of these.

**Conclusions:**

NCCP15736 and NCCP15737 differ at the genomic level, even though they share features such as virulence-related genes. NCCP15737 has a deletion in *fimD,* which may underlie its asymptomatic character. We conclude that complete genome sequencing and integration of other types of omics data are needed to fully reveal the mechanism underlying the asymptomatic character of NCCP15737.

**Electronic supplementary material:**

The online version of this article (doi:10.1186/s13099-016-0138-9) contains supplementary material, which is available to authorized users.

## Background

*Escherichia coli* is a typical member of the normal microflora of the human gastrointestinal tract [1]. However, some *E. coli* isolates cause serious disease. They can be divided into three major subgroups: commensal or nonpathogenic strains, pathogenic strains that cause intestinal infection, and extraintestinal pathogenic strains [2]. Intestinal pathogenic *E. coli* include enteroaggregative *E. coli*, enterohemorrhagic *E. coli* (EHEC), enteropathogenic *E. coli* (EPEC), enteroinvasive *E. coli*, and enterotoxigenic *E. coli* (ETEC). Shiga toxin-producing *E. coli* (STEC) O157:H7 in humans was first reported in 1983 [3–5]. STEC causes a variety of diarrheal diseases and hemolytic uremic syndrome (HUS) [6]. EHEC belongs to the STEC group but it is associated with a distinctive clinical syndrome, namely hemorrhagic colitis (HC), mainly caused by *E. coli* O157:H7 [7, 8]. Shiga toxin (Stx) inhibits protein synthesis by disrupting the 28S RNA of the 60S ribosomal subunit [9]. Shiga toxins can be classified into two groups: Stx1 and Stx2 [7]. Stx1 originates from *Shigella dysenteriae* and there are three subtypes: Stx1a, Stx1c and Stx1d; these genes are highly conserved in STECs. Stx2 shows a lower degree of conservation and includes several variants: Stx2a, Stx2b, Stx2c, Stx2d, Stx2e, Stx2f, and Stx2g [10]. Most outbreaks involve STEC O157:H7, but outbreaks caused by non-O157 STEC have shown a recent increase [11]. Thus, a better understanding of the causes of the asymptomatic character of STEC strains is required. Non-O157 STEC includes the O8:H, O26:H, I26:H11, O91:H21, O103:H2, O111:H, O113:H21, O128:H2, and O145:H [7] serotypes.

Two STEC O91:H21 isolates were used in this study, one from a symptomatic carrier and one from an asymptomatic carrier, both isolated during a recent outbreak in Korea [12]. Molecular and cellular analyses to investigate differences in pathogenicity between the isolates were performed in a previous study. A reduced adherence phenotype and transcriptional repression of type I fimbriae genes were identified in the isolates from the asymptomatic carrier; these two factors may explain why the isolates cause no symptoms. However, the mechanism underlying the transcriptional repression of type I fimbriae is not yet understood at the genomic level. To investigate the differences between the O91:H21 isolates from symptomatic and asymptomatic carriers and to explore the genetic basis underlying these differences, whole-genome sequencing and comparative genomic analyses were performed.

## Methods

### Strain, isolation, and serotyping

An outbreak of STEC at an elementary school was reported in Gwangju, Korea on July 2004 [12]. A total of 1643 stool samples were obtained from asymptomatic individuals and all isolates were biochemically characterized using the API20E system (Biomerieux, Marcy l’Etoile, France). A total of 74 STEC isolates were characterized as positive for STEC but caused no symptoms. Apart from the isolates from asymptomatic carriers, one STEC isolate from a symptomatic carrier was characterized. The isolated strains were deposited in the National Culture Collection for Pathogens (NCCP) at the Korean National Institute of Health under accession numbers NCCP15736 and NCCP15737. For the present study, NCCP15736 and NCCP15737 were obtained from the NCCP for whole-genome sequencing. This research has been reviewed and approved by the Institutional Review Board of the Korean Centers for Disease Control and Prevention.

### Library preparation and whole-genome sequencing

A sequencing library was constructed using the TruSeq Sample Preparation Kit (Illumina, San Diego, CA, USA) following the manufacturer’s instructions. Genomic DNA was end repaired and ligated with paired-end sequencing adapters. DNA fragments with the desired length of ~500 bp were selected by gel electrophoresis. A sequencing library was produced by PCR amplification. The Illumina HiSeq 2000 platform was used for whole-genome sequencing.

### Genome assembly and annotation

Low-complexity reads, reads with quality scores <Q20, adapter sequences, and duplicate reads were discarded. De novo assembly of high-quality reads was performed with SOAPdenovo (version 1.05) [13]. The de novo assembly results were corrected based on alignment of all reads that passed the quality control threshold against the assembly results using SOAPaligner (version 2.21) [14]. After correction, scaffolds >500 bp in length were considered for downstream analysis.

Open reading frames and annotated open reading frames were identified using the Rapid Annotation using Subsystem Technology (RAST version 4.0) [15] server pipeline. The coding sequences (CDSs) of NCCP15736 and NCCP15737 were compared using the sequence base comparison functionality of the RAST server. For comparison of type I fimbriae gene clusters between the two strains, the sequence base comparison functionality of the RAST server was also used. To investigate the virulence factor genes, a BLAST search of the total open reading frames (ORFs) of NCCP15736 and NCCP15737 against the virulence factor genes of *E. coli* listed in VFDB [16] was performed with an e-value threshold of 1e − 5. To select homologous virulence factor genes, the BLAST Score Ratio (BSR) was calculated and only genes with a BSR score ≥0.4 were used in further analyses. The BSR score was calculated using our in-house scripts. We excluded genes with coverage lower than 60%, even if they showed high sequence identity. Phage-associated gene clusters in the genome sequences of NCCP15736 and NCCP15737 were identified using the PHAST server [17]. Three scenarios for the completeness of the predicted phage-associated regions were defined according to how many genes/proteins of a known phage the region contained: intact (≥90%), questionable (90–60%), and incomplete (≤60%).

### Phylogenetic analysis and genomic structure comparison

To infer the evolutionary relationships among *E. coli* O91, including NCCP15736 and NCCP15737, multiple sequence alignments of the whole genome were performed with Mugsy (version 1.2.3) [18]. The generalized time-reversible [19] + CAT model [20] was used to infer the structure of maximum-likelihood phylogenetic trees using FastTree (version 2.1.7) [21]. FigTree (version 1.3.1) (http://tree.bio.ed.ac.uk/software/figtree/) was employed for tree visualization. For comparison of genomic structures between the two strains, the progressive alignment algorithm in Mauve (version 2.3.1) [22] was used. The BLAST algorithm was used to compare phage-associated regions.

### Quality assurance

The genomic DNA was purified from a pure culture of a single bacterial isolate of NCCP15736 and NCCP15737, respectively. Potential contamination of the genomic library by other microorganisms was assessed using a BLAST search against the non-redundant database. We also checked for contamination by other genomes by confirming coverage distribution.

## Results and discussion

### General features

**Fig. 1:**
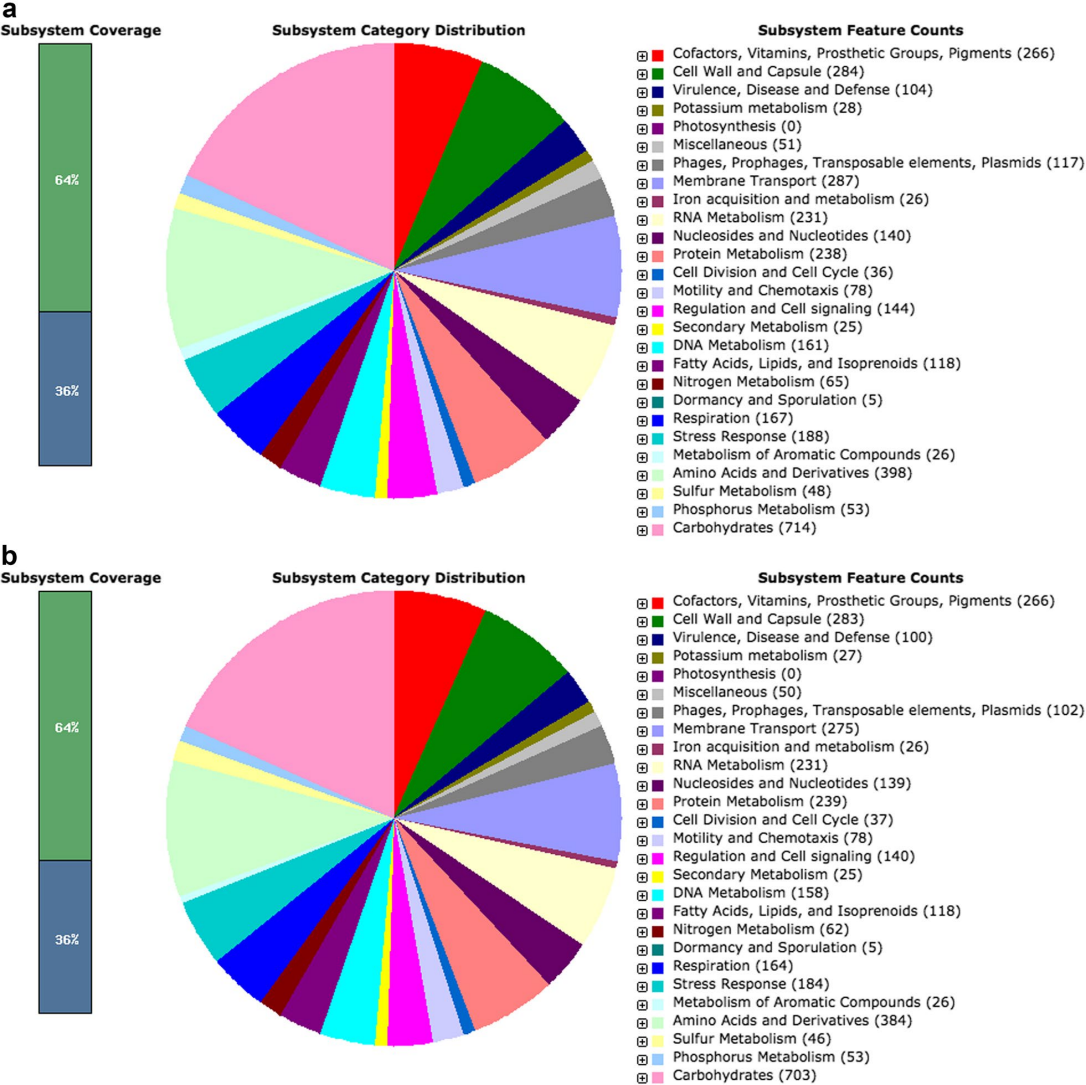
Subsystem category distribution of NCCP15636 and NCCP15737 based on the SEED databases. **a** NCCP15736, **b** NCCP15737

A total of 569,860,000 bp and 576,270,000 bp of paired-end reads were generated with the Illumina HiSeq 2000 platform from genomic DNA of NCCP15736 and NCCP15737. We used 517 Mbp and 477 Mbp of high-quality reads for assembly after quality control. After de novo assembly, a total of 151 scaffolds with a scaffold N50 of 133,815 bp were obtained for NCCP15736 and 156 scaffolds with a scaffold N50 of 140,358 bp were assembled for NCCP15737. The draft genome size of NCCP15736 was 5079,147 bp and that of NCCP15737 was 5,126,930 bp. The genomic features of NCCP15736 and NCCP15737 are summarized in Table 1. Based on a RAST analysis, 4823 putative CDSs and 14 tRNA genes were identified in the NCCP15736 genome. A total of 4924 putative CDSs and 26 RNAs were identified in the NCCP15737 genome (Fig. 1; Additional file 1: Table S1).

**Table 1:** Genomic features of NCCP15736 and NCCP15737 strains of *Escherichia coli*

Strain	NCCP15736	NCCP15737
Genome (Mb)	5.08	5.13
% GC	50.59	50.62
Total open reading frames	4823	4924
tRNAs	14	22
rRNAs	0	4

### Comparison of genome structure

**Fig. 2:**
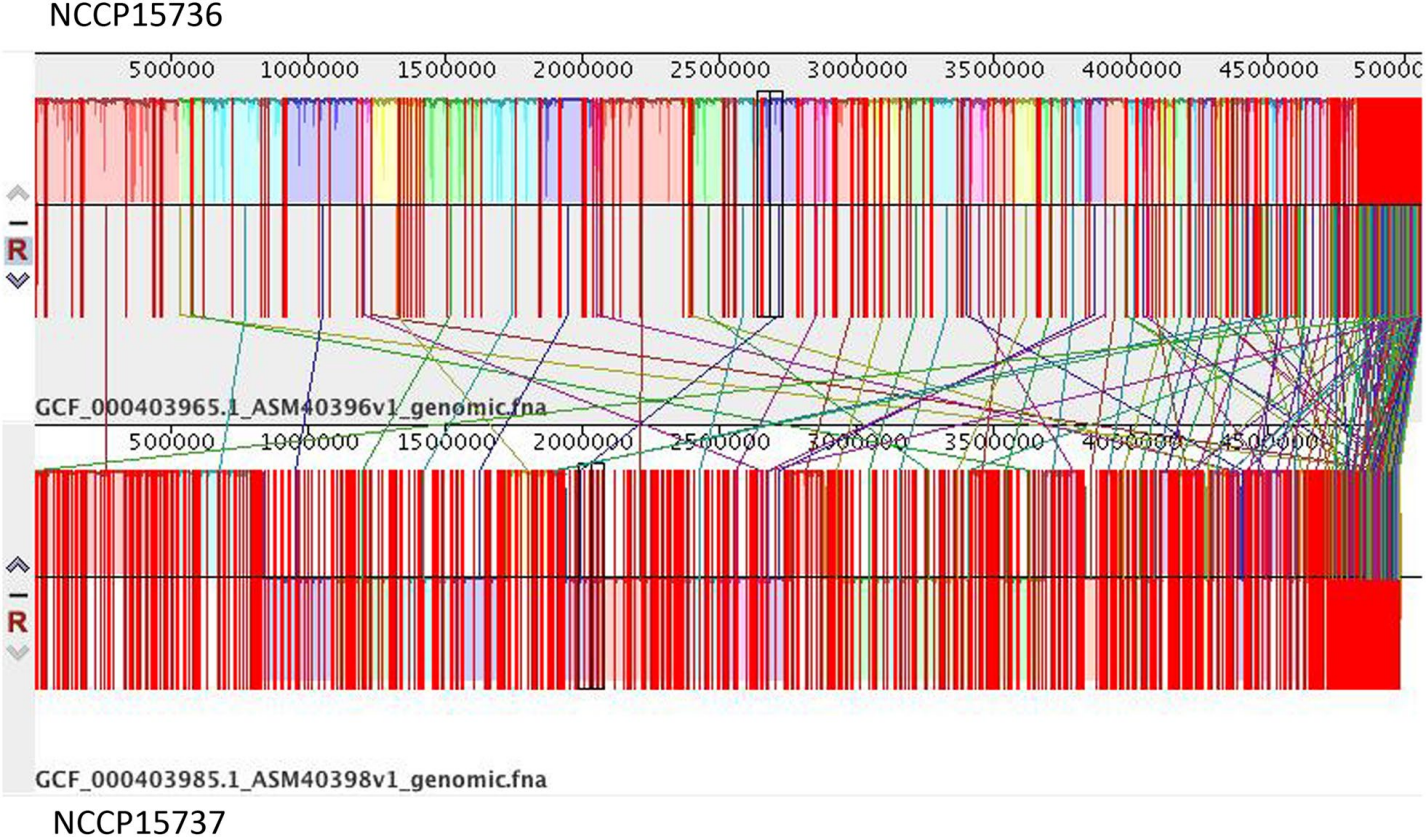
Comparative analysis of genomic structure of strains NCCP15736 and NCCP15737 of *Escherichia coli*. Comparison of genome structure between NCCP15736 and NCCP15737 using a progressive alignment implemented in Mauve

In the comparative analysis of genomic structure performed using the progressive alignment function of Mauve, we detected structures that were highly conserved between NCCP15736 and NCCP15737 (Fig. 2). Several unaligned scaffolds were also detected.

### Phylogenetic analysis

**Fig. 3:**
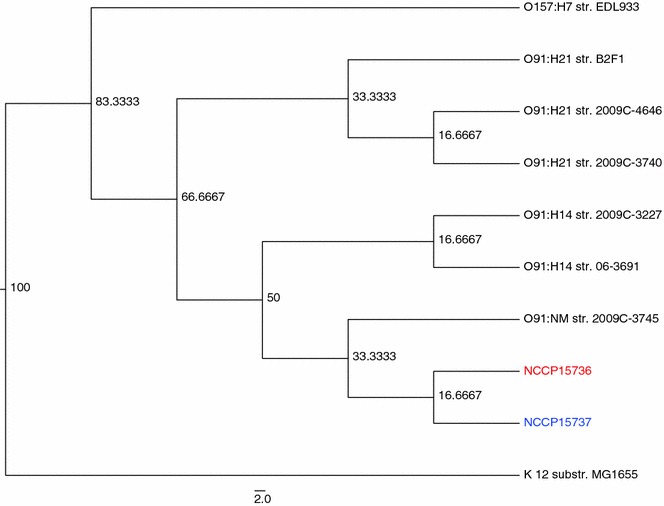
Whole-genome based phylogenetic tree of NCCP15736 and NCCP15737 strains of *Escherichia coli*. Evolutionary time is scaled by 100; lower values imply relatively recent branching. The *scale* indicates the number of substitutions per site. The NCCP15736 (*red*) and NCCP15737 (*blue*) strains were placed in the same clade. The isolate most closely related to the two strains, based on whole-genome phylogenetic analysis, was *E. coli* O91:NM str. 2009C-3745

Phylogenetic comparison of candidate genes, implemented in SEED [23], showed that NCCP15736 and NCCP15737 are most close to *E. coli* O104:H4 str. GOS1 (score 516). A whole-genome phylogenetic tree showed that NCCP15736 is close to NCCP15737 and that both strains are closer to *E. coli* O91:NM str. 2009C-3745 (Fig. 3).

### Type I fimbriae operon

**Fig. 4:**
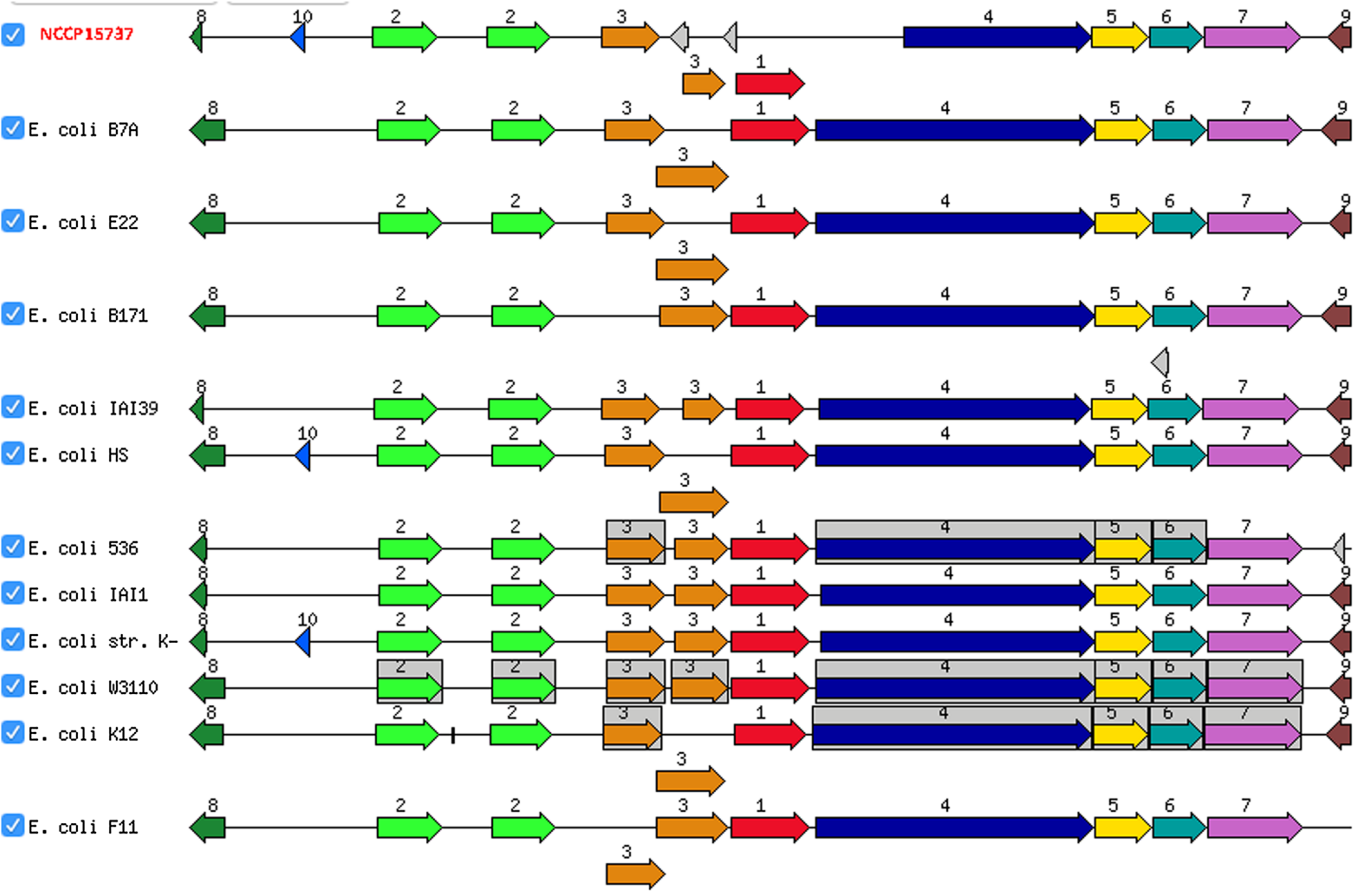
Comparative map of type I fimbriae in the NCCP15736 genome and other closely related species. Nine genes were highly conserved in the eleven strains, and only *fimD* (*4*) was truncated in NCCP15736.* Numbers* indicate genes encoding the following proteins: chaperone FimC (*1*); type I fimbriae regulatory proteins FimB and FimE, from *left to right* (*2*); type I fimbriae major subunit FimA (*3*); type I fimbriae anchoring protein FimD (*4*); type I fimbriae adaptor subunit FimF (*5*); type I fimbriae adaptor subunit FimG (*6*); mannose-specific adhesion FimH (*7*); N-acetylneuraminic acid outer membrane channel protein NanC (*8*); fructuronate transporter GntP (*9*).* Gray background boxes* indicate that the genes in the relative positions are conserved in at least four species. The comparative map was created with the genome browser of the SEED viewer (version 2.0)

In a previous study, it was reported that the cell surface of NCCP15737 is completely bald; the lack of type I fimbriae was concluded to be the main cause of the asymptomatic character of NCCP15737 [12]. From the comparison of NCCP15736 and NCCP15737 using the sequence-based comparison functionality of the RAST server, we determined that *fimD* is truncated in NCCP15737, and its product is 591 amino acids instead of the full 852 amino acids (Fig. 4). The product of *fimD* is also known as fimbrial usher protein, which anchors the type I pilus to the cell surface [24]. Type I fimbriae are important for the virulence and survival of *E. coli* [25]. To investigate the role of *fimD* in the infection and pathogenicity of *E. coli* O91:H21, further experiments such as a *fimD* deletion study and microarray analysis of gene expression in NCCP15736 and NCCP15737 are required.

### Virulence factors

NCCP15736 was isolated from a symptomatic human carrier but NCCP15737 was isolated from an asymptomatic human carrier. To determine the causal mechanisms underlying the observed pathogenicity, we investigated the virulence factors of NCCP15736 and compared these factors with those of NCCP15737. Using a BLAST search against VFDB, we identified the same number, 25, of virulence factors from NCCP15736 and NCCP15737, respectively (Additional file 2: Table S2). The 25 virulence genes present in NCCP15736 were also present in NCCP15737. The virulence genes of NCCP15736 and NCCP15737 can be classified into five categories: adherence, invasion, iron uptake, secretion system, and toxins. In the adherence category, *E. coli* common pilus (ECP)-related genes (*ecpA, B, C, D, E*, and *ecpR*) F1C fimbriae (*foc*C), and type I fimbriae genes (*fimA, B, C, D, E, F, G, H*, and *I*) were identified. Tia invasion determinant (*tia*) [26], which belongs to the invasion category and originates from *E. coli* O1:K1, was identified in both strains. In the iron uptake category, iron-regulated element gene (*ireA*) and salmochelin siderophore-related gene (*iroN*) were identified in NCCP15736 and NCCP15737. Neither strain contained all of the genes in the LEE-encoded TTSS effectors category, harboring only one secretion gene, *escR* [27]. In the toxins category, alpha-hemolysin–related genes (*hlyA, B* and *D*) [28] were identified. Alpha-hemolysin is a major virulence factor present in ETEC, STEC, and EPEC strains. It is acquired by horizontal gene transfer via conjugative plasmids [29]. Shiga-like toxin-related genes (*stx1A* and *1B*) [30] were present in both of the strains and exhibited 100% sequence conservation. In summary, the NCCP15736 and NCCP15737 strains showed the same virulence factors, although NCCP15736 was isolated from a symptomatic carrier and NCCP15737 was isolated from an asymptomatic carrier. In a previous report [12], the expression of type I fimbriae genes was found to be significantly repressed, and the repression was hypothesized to be the main cause of the asymptomatic nature of NCCP15737.

### Phage-associated regions

Prophages are mobile genetic elements that can deliver antimicrobial-resistance genes [31] or virulence factors [32] to bacterial hosts and contribute to the diversity of host genomes [33]. We identified ten phage-associated regions (S1–S10) in the NCCP15736 genome and nine phage-associated regions (A1–A9) in the NCCP15737 genome using the PHAST algorithm (Additional file 3: Table S3). Seven of the ten phages in NCCP1576 were intact, and seven of the nine phages in NCCP15737 were intact. NCCP15736 and NCCP15737 each contain two incomplete prophages. Only one questionable prophage, in the S6 region (Stx2-converting phage 1717), was identified in the NCCP15736 genome. Five of the identical phage-associated regions, as determined via a BLAST search, were shared by the two strains. The prophage-associated regions S2, S6, S8, S9, and S10 were unique to NCCP15736, and the A5, A7, A8, and A9 regions were unique to the NCCP15737 genome.

## Future directions

The number of outbreaks caused by non-O157 STEC has increased recently and is causing growing concern. In this study, we performed whole-genome sequencing and comparative genomic analysis of two strains, NCCP15736 and NCCP15737. Our whole-genome sequencing and bioinformatics analyses revealed that NCCP15736 and NCCP15737 have the same virulence gene profiles, but NCCP15737 *fimD* shows a deletion. Even though our results did not reveal the genomic basis of the transcriptional repression of type I fimbriae genes in NCCP15737, we provided a structural basis for the relationship between the deficiency in the gene encoding type I fimbriae and the asymptomatic character of NCCP15737. We suggest that complete genome sequencing and integration of other types of omics data are required to fully reveal the mechanism underlying the asymptomatic character of NCCP15737.

## Additional files

**Additional file 1.** Annotated genes of NCCP15736 and NCCP15737 using the RAST server.

**Additional file 2.** Virulence genes of NCCP15736 and NCCP15737.

**Additional file 3.** Phage-associated regions in NCCP15736 and NCCP15737.

## Abbreviations

CDS: coding DNA sequences
EHEC: enterohemorrhagic *Escherichia coli*
NCCP: National Culture Collection for Pathogens
RAST: Rapid Annotation using Subsystem Technology
STEC: Shiga toxin-producing *Escherichia coli*
str.: strain
substr: substrain
